# Y chromosome microdeletion and cytogenetic findings in male infertility: A cross-sectional descriptive study

**DOI:** 10.18502/ijrm.v19i2.8473

**Published:** 2021-02-21

**Authors:** Meenakshi Arumugam, Deyyanthody Prashanth Shetty, Jayarama Shanker Kadandale, Suchetha Nalilu Kumari

**Affiliations:** ^1^KSHEMA Centre for Genetic Services, Kowdur Sadananda Hegde Medical Academy, Nitte University, Mangalore, Karnataka, India.; ^2^Department of Biochemistry, Kowdur Sadananda Hegde Medical Academy, Nitte University, Mangalore, Karnataka, India.

**Keywords:** Chromosome aberrations, Infertility, Chromosome deletion, Polymerase chain reaction, Sequence tagged sites.

## Abstract

**Background:**

Infertility affects about 15% of couples worldwide, and the male factor alone is responsible for approximately 50% of the cases. Genetic factors have been found to play important roles in the etiology of azoospermia and severe oligospermia conditions that affect 30% of individuals seeking treatment at infertility clinics.

**Objective:**

To determine the frequency of chromosomal abnormalities and Y chromosome microdeletion in infertile men.

**Materials and Methods:**

A total of 100 infertile men with abnormal semen parameters were included in this study from 2014 to 2018. Chromosomal analysis was carried out using standard G-banding using Trypsin Giemsa protocol. Multiplex polymerase chain reaction was used to determine the Y microdeletion frequency.

**Results:**

All participants were aged between 22 and 48 yr with a mean and standard deviation of 35.5 ± 5.1. Of the 100 subjects included in the study, three had Klinefelter syndrome-47,XXY, one had balanced carrier translocation-46,XY,t(2;7)(q21;p12), one with the balanced carrier translocation with inversion of Y chromosome 45,XY,der(13;14)(q10;q10),inv(Y), one had polymorphic variant of chromosome 15, one had Yqh-, and another had an inversion of chromosome 9. Y chromosome microdeletion of Azoospermia factor c region was observed in 2% of the cases. To the best of our knowledge, the current study is the first reported case with unique, balanced carrier translocation of chromosome 2q21 and 7p21.

**Conclusion:**

The present study emphasizes the importance of routine cytogenetic screening and Y microdeletion assessment for infertile men, which can provide specific and better treatment options before undergoing assisted reproductive technology during genetic counseling.

## 1. Introduction

Infertility is a disease of the reproductive system and the incapability of a couple at reproductive age to conceive naturally after 12 months or more of regular unprotected sexual intercourse (1). Infertility affects about 15% of couples worldwide, and men are accountable for around 50% of the cases. The leading causes of infertility in men are abnormal semen parameters such as azoospermia, oligozoospermia, asthenozoospermia, teratozoospermia, and oligoasthenoteratozoospermia, which occur during spermatogenesis (2).

Spermatogenesis is a complex process involving nearly 2,300 genes in the formation and regulation of spermatozoa (3). Based on systematic cytogenetic studies carried out on male infertility, it is estimated that the incidence of chromosomal aberrations is about 5.8% (4). Chromosomal abnormalities can be numerical (gain or loss of a chromosome) or structural (balanced or unbalanced chromosome) and involve both sex chromosomes and autosomes.

Y chromosome structural abnormalities like deletion, ring chromosome, and isochromosome may lead to different phenotypes. Deletion of the sex-determining region located in the p arm of the Y chromosome directly disturbs the testis differentiation, which leads to phenotypically female with streak gonads, while the Y microdeletions especially involved with azoospermia factor (AZF) a, b, and c regions on the q arm of the Y chromosome lead to male infertility (5). The incidence of deletions on the Y chromosome was estimated to be 7-8% with a higher deviation (6).

Severe oligozoospermia and azoospermia with normal karyotype have been reported in 10-15% of the infertile males, involving deletion of the deleted in azoospermia gene of the AZFc region (7).

Homologous recombination amongst Y-specific repeats generates deletions, which leads to spermatogenic failure due to the removal of AZF functional genes (8). The Y microdeletion frequency fluctuates from 0.9- 55.5% in various studies conducted on infertile male. The recommended sequence-tagged sites (STS) markers by the European Academy of Andrology (EAA) are the only six to find out Y microdeletion in infertile male with a higher detection rate of almost 90% (9). However, the recommendation of Y microdeletion to infertile men for their routine clinical practice has not been adopted regularly.

This study has been conducted to determine the frequency of chromosomal abnormalities and develop a molecular-level diagnosis of male infertility and to standardize the Y microdeletion protocol.

## 2. Materials and Methods

This cross-sectional descriptive study was performed on 100 infertile men with abnormal semen parameters such as azoospermia, oligozoospermia, teratozoospermia, and asthenozoospermia, referred to the KSHEMA Centre for Genetic Services (K. S. Hegde Medical Academy, Mangalore, India) between 2014 and 2018. The studied participants were aged between 22 and 48 yr with a mean and standard deviation of 35.5 ± 5.1 yr. Infertile men with azoospermia, oligospermia, teratospermia and asthenozoospermia were included in this study. Patients who were not willing to participate and having normal sperm count were excluded. Peripheral blood (2 ml) was collected from all participants in heparin and EDTA vacutainer for chromosomal analysis and Y microdeletion detection, respectively.

### Chromosomal analysis

Chromosomal analysis was carried out by the standard G-banding protocol (10) with minor modifications. The culture was set up in a 15-ml centrifuge tube with 500-µl whole blood and 5 ml PB - Peripheral blood max media (Gibco USA). Phytohemagglutinin (Phytohemagglutinin Form M: Gibco USA), 300 µl was added to the culture as a mitogen and incubated at 37°C in 5% CO${}_{2}$ (Panasonic) for 68 hr.

Colcemid (0.08 µg/ml) (Gibco, USA) 45 µl was added to arrest the cells at the metaphase stage at the 68^th^ hr.

Next, 9 ml of 0.075 M KCl (Merck, India) was added and incubated at 37°C for 13 min. Then, the cells were fixed by using a 3:1 ratio of methanol (LOBA Chemie, India) and acetic acid (Merck, India) (Carnoy's fixative). The cell pellet was dropped on slides and aged overnight at 60°C hot air oven (Ascension Innovation) overnight. G-banding using Trypsin Giemsa was done by treating with 0.05% trypsin and staining with 4% Giemsa stain.

Twenty well-spread metaphases with excellent band resolution were selected for analysis under oil immersion (100×) using a bright field microscope (BX53, Olympus, Japan).

Karyotypes were reported using the GENASIS Software (Version 7.2, Applied Spectral Imaging, Israel). Identified chromosomal abnormalities were designated as per the International System for Human Cytogenetic Nomenclature 2013 guidelines (11).

### Y Microdeletion

Genomic DNA was extracted by a phenol-chloroform manual method from peripheral blood leukocytes. A series of 10 STS markers were analyzed by multiplex polymerase chain reaction (PCR) using commercially available primers (Invitrogen in Life technologies). The STS markers used in this study for AZFa are sY84, sY86; for AZFb sY127, sY134, and sY143; for AZFc sY156, sY158, sY254, and sY255.

Microdeletions on AZFa, AZFb, and AZFc were carried out using the multiplex PCR technique. The Y microdeletion of genomic DNA was standardized based on Simoni *et al* guidelines with modifications (12).

Also, an additional primer was used to amplify the ZFY regions as internal controls. Table I shows the primer sequences used and the size of related PCR products.

Along with each set of primers as an internal control, we ran one female DNA sample, one normal male DNA, and water as a substitute of DNA every time. The genomic DNA (100 ng/μl) was added to a mixture of 50 mM MgCl${}_{2}$, 100 mM each dNTP, 100 μmol of primer pairs, and 1 unit of Taq DNA polymerase (Invitrogen, Life technologies, USA), adjusted to a final volume of 25 μl. PCR program was carried out on a Bio-Rad Thermocycler (USA). Initial denaturation was carried out at 95°C for 10 min, followed by 35 cycles of 94°C for 30 sec denaturation and annealing at 58°C for 90 sec with 1-min extension at 72°C. For Multiplex B, C1 and C2, 94°C for 5 min as initial denaturation, continued with 35 cycles of 94°C for 30 sec, annealing at 58°C for 30 sec, and extension at 72°C for 30 sec was carried out. All PCR amplification products were subjected to agarose (2%) gel electrophoresis of 100 V current by using 1X Tris base, acetic acid, and EDTA buffer, with Ethidium bromide along with 100 bp DNA ladder and visualized under ultraviolet light using the Geldoc (BIORAD - GelDoc EZ Imager, USA). If any deleted specific STS primer bands were noticed, then it was considered that the associated region was deleted.

**Table 1:** List of STS markers and primer sequences used for detection of Y chromosome microdeletion


**STS**	**Region**	**Primer sequence**	**Product size (bp)**
**sY84**	AZFa	5'AGA AGG GTC TGA AAG CAG GT3' 5'GCC TAC TAC CTG GAG GCT TC3'	326
**sY86**	AZFa	5'GTG ACA CAC AGA CTA TGC TTC3' 5'ACA CAC AGA GGG ACA ACC CT3'	320
**sY127**	AZFb	5'GGC TCA CAA ACG AAA AGA AA3' 5'CTG CAG GCA GTA ATA AGG GA3'	274
**sY134**	AZFb	5'GTC TGC CTC ACC ATA AAA CG3' 5'ACC ACT GCC AAA ACT TTC AA3'	301
**sY143**	AZFb	5'GCA GGA TGA GAA GCA GGT AG3' 5'CCG TGT GCT GGA GAC TAA TC3'	311
**sY156**	AZFc	5'AGG AAC TGG CAG GAT TAG CC3' 5'ATG TCA GGG TTT CCT TTG CC3'	950
**sY158**	AZFc	5'CTC AGA AGT CCT CCT AAT AGT TCC3' 5'ACA GTG GTT TGT AGC GGG TA3'	231
**sY254**	AZFc	5'GGG TGT TAC CAG AAG GCA AA3' 5'GAA CCG TAT CTA CCA AAG CAG C3'	400
**sY255**	AZFc	5'GTT ACA GGA TTC GGC GTG AT3' 5'CTC GTC ATG TGC AGC CAC3'	126
**Internal Positive ZFY**	Yp11.3	5'ACC RCT GTA CTG ACT GTG ATT3' 5'GCA CYT CTT TGG TAT CYG AGA3'	495
All primers were non-polymorphic short DNA fragments, and we designed them into four sets as multiplex A, B, C1, and C2. Multiplex A: 4 Primers - ZFY, sY86, sY127, sY254, Multiplex B: 4 Primers - ZFY, sY84, sY134, sY255, Multiplex C1: 2 Primers - sY143, sY158, Multiplex C2: 1 Primer - sY156			

### Ethical considerations

The ethical clearance for this study was obtained from the institutional ethics committee (Ref. No. NU/CEC/Ph.D-19/2014), and informed consent was obtained from all participants before the study.

### Statistical analysis

The collected information was summarized using frequency/percentage for qualitative data and mean with standard deviation for quantitative data. The frequency was used to determine the chromosomal analysis and Y microdeletion. Data management and analysis were performed using Microsoft Excel and Statistical Package for the Social Sciences, version 16.0.1 (SPSS Inc., Chicago, IL, USA).

## 3. Results

Of the 100 participants, 68% were infertile men with azoospermia, 18% with oligospermia, 6% with severe oligospermia, and 8% with oligoasthenoteratozoospermia. All of them had chromosomal analysis, of which 37 cases were reported earlier (13), and in the remaining 63 are described in the present study.

### Determination of chromosomal abnormalities frequency

**Figure 1:**
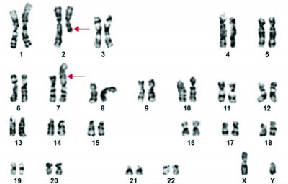
Karyotype with balanced translocation of chromosomes 2 and 7 - 46,XY,t(2;7)(q21;p21).

**Figure 2:**
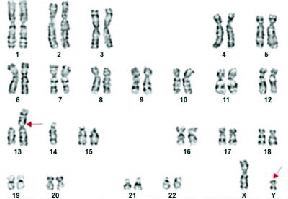
Karyotype with Robertsonian translocation of chromosomes 13,14 and inversion of Y chromosome-45,XY,der(13;14)(q10;q10),inv(Y)(q11.2).

Of the 63 cases described in the current study, normal karyotype of 46,XY was observed in 53 cases, chromosomal abnormalities of 47,XXY-Klinefelter syndrome in 3 cases, balanced carrier translocation of 46,XY,t(2;7)(q21;p12) (Figure 1) in 1 infertile man with severe oligospermia, 45,XY,der(13;14)(q10;q10),inv(Y) (Figure 2) in another case with oligospermia, polymorphic variants of chromosome Y- inv(Y) in 2 cases, inversion of chromosome 9 in 2 cases, and 15ps+ in one case (Table II).

### Determination of Y-microdeletion frequency

Using 10 STS markers in 100 infertile men detected only two infertile males (2%) with deletion of AZFc region on Yq11. Y microdeletion detection was negative in 98% of the cases. The tested four specific STS markers sY156, sY158, sY254, sY255 for AZFc region on the Y chromosome was absent in one azoospermic male and one severe oligospermic male.

**Table 2:** Chromosomal abnormalities observed in infertile men


	**Karyotype**	**Cases [n (%)]**
**No chromosomal abnormalities**	46,XY	53 (84)
**Numerical abnormalities **	47,XXY	3 (4.8)
**Balanced carrier translocation**	46,XY,t(2;7)(q21;p12)	1 (1.6)
	45,XY,der(13;14)(q10;q10),inv(Y)	1 (1.6)
**Polymorphic variant of chromosome 15**	46,XY,15ps+	1 (1.6)
**Polymorphic variant of chromosome Y**	46,XY,qh-	2 (3.2)
**Inversion of chromosome 9**	46,XY,inv(9)(p11q13)	2 (3.2)

## 4. Discussion

Specific chromosomal abnormalities like balanced carrier translocations and Y microdeletions on the AZF region are the reason for the many infertile males, but not always practiced routinely. Failure to screen genetic factors such as karyotyping and Y microdeletion leads to unsuccessful assisted reproductive technology (ART) procedure, and there will be a chance to transfer the same abnormality to the offspring. Specifically, balanced carrier translocations cause the production of unbalanced spermatozoa and offspring with mental retardation, whereas Y microdeletion leads to offspring with the same problem of infertility, especially when the offspring is male.

The last 20 yr of research has indicated that various chromosomal abnormalities caused by the interruption of spermatogenesis in different stages end up with infertility. Former studies have shown a high prevalence of chromosomal abnormalities eventually in infertile azoospermic men with sex chromosome abnormalities than oligozoospermic patients with frequent autosomal abnormalities (14). In the present study, we observed sex chromosomal abnormalities and autosomal abnormalities in azoospermic men and reported only one oligozoospermic male with balanced carrier translocation.

Chromosomal analysis is important in identifying the cause of Klinefelter's syndrome with the phenotypic features of tall stature, testicular atrophy, azoospermia, and infertility with a prevalence of 0.1% in the general population (15). In the present study, we observed three azoospermic men with Klinefelter syndrome - 47,XXY. The extra X chromosome of Klinefelter syndrome influences the mechanism of spermatogenesis, which affects testicular development, Leydig cell insufficiency, Sertoli, and Leydig cells apoptosis regulation (16). Non-disjunction of sex chromosomes is the primary genetic cause for the Klinefelter syndrome, which leads to the presence of extra X chromosomes. The occurrence of the additional X chromosome by maternal during oogenesis is likely to occur from nondisjunction in meiosis I or II while in paternal (spermatogenesis) can only be derived from nondisjunction in meiosis I (17). Klinefelter syndrome is always diagnosed when a person undergoes fertility assessment not at their earlier age.

Balanced reciprocal translocation carriers have a higher risk of producing unusual pregnancies with unbalanced chromosomal aberrations by precise functional roles of genes at particular breakpoints in spermatogenesis (18). A detailed molecular analysis of the translocated region is needed because of the possible cause of this region's involvement in male infertility due to disruption of meiosis (19). Infertility cases with various balanced translocations of different chromosomes such as t(1;19), t(3;13), t(1;9), t(9;10), t(9;3), t(1;4), t(7;8), t(3;6), t(1;11), t(1;10), t(3;18), t(7;9); t(7;14), t(7;17), (13;19), t(6;17) have been reported earlier (20, 21). According to the existing studies, 7q31 and 7q36 breakpoints are related to pre-gestational infertility, whereas 7q21.2, 7q22, and 7q32 breakpoints are associated with gestational infertility (22). However, whichever category of infertility is involved with chromosome 7 must be counseled based on specific breakpoints.

Further, balanced carrier translocation of 2p1;7q32, 2p13;7q34, 2q31;7q34, 2p23;7p22 have also been reported earlier (23). Based on our knowledge, 2q21;7p21 was observed for the first time in the present study. Infertile men with carriers of der(13;14) frequency were approximately 10-fold higher than a newborn survey with missed abortions, stillbirths, and offspring with a congenital malformation (24). While most of the Robertsonian translocations are inherited from paternal, nearly 40% can be de novo translocations due to rearrangements in meiosis (25). However, in our study, we observed a de novo translocation of 45,der(13;14) with inversion Y in one azoospermic male, which is not a frequent case ever reported earlier.

Most infertility cases with inversion Y have not influenced any of the phenotypic features, however, a del(Y)(q11.1-q11.2) or disruption of the deleted in azoospermia genes caused by pericentric inversion has been reported in one male infertility case (20). The 46,XY,qh- karyotype is considered as a normal polymorphic variant, which occurs in the general population. A study conducted by Pan *et al* (26) with 46,XY,qh- cases perceived 64.7% of patients with AZF microdeletion and insisted that Y microdeletion screening is a must in such type of cases. In the present study, we have observed 2% of the patients with 46,XY,qh- and their Y microdeletion results were normal. Inversions occur when a single chromosome undergoes two breaks and is reconstructed with the segment between the breaks inverted during meiosis, which can be either pericentric or paracentric. The heterochromatin polymorphisms of autosomes (chromosomes 1, 9, 16 and Y) reported by earlier studies revealed no phenotypic or clinically adverse effects or any apparent association with infertility (27).

A study conducted in Korea with 1,226 infertile males showed the overall frequency of Y chromosome microdeletions in 10.93% (134/1,226) patients, of which the AZFc region was the most frequent with 51.49% (69/134), followed by the AZFb with 8.21% and AZFa with 7.46% (28). A study conducted in Japan with 162 infertile males showed seven (4.3%) cases were positive for Y microdeletion (29). Two other studies conducted in Egypt showed 4 out of 100 infertile men (4%) (30), and 0/40 infertile males (0%) (31) had absence of AZFc region on the Y chromosome. Furthermore, a study performing Y-chromosome screenings on 1,387 infertile men in Portugal reported that AZF microdeletions in 128 cases: 17 (1.2%) in AZFa, 26 (1.9%) in AZFb, and 85 (6.1%) in AZFc (32). A study conducted on 1,636 infertile subjects in Mumbai showed 3.4% (56/1,636) of the cases had Yq microdeletions (6). Various Indian studies listed in Table III with different STS markers used for Y microdeletion showed different frequency of Y microdeletion.

Variation among Y microdeletion frequency was due to the selection of STS markers, inclusion and exclusion criteria of studied participants, and predominantly their ethnicity. In the present study, only 2% of Y microdeletion was detected, which indicates that the southern-region population had a lower Y microdeletion frequency. Along with EAA-recommended STS markers, each ethnicity population has to standardize with additional markers to increase the Y microdeletion detection rate (9). A study conducted by Rani and colleague (33) illustrated a high frequency of deletion (29.4%) in the AZF region and concluded that ethnicity plays a significant role in Indian populations. Chromosomal abnormalities and Y microdeletion have emerged as a leading cause of male infertility. The strength of our dataset is the reliability of the results due to homogeneous procedures carried out.

**Table 3:** Studies on Y chromosome microdeletions in different regions of India


**Author (ref)**	**Place **	**Total studied subjects **	**Positive for Y microdeletion [No. (%)]**	**Total studied STS markers **	**Number of EAA STS markers **	**EAA STS markers deletion (%)**
**Thangaraj ** ***et al.*** ** (34)**	Kolkata	340	29 (8.5)	32	6	6.5
**Swarna ** ***et al.*** ** (35)**	Hyderabad	50	4 (8.0)	2	1	2
**Athalye ** ***et al.*** ** (36)**	Mumbai	100	12 (12.0)	18	2	6
**Rao ** ***et al.*** ** (37)**	Hyderabad	251	10 (3.9)	17	5	1.5
**Kumar ** ***et al.*** ** (38)**	New Delhi	109	7 (8.5)	8	6	8.5
**Sachdeva ** ***et al.*** ** (9)**	New Delhi	200	21 (10.5)	25	6	3
**Mahanta ** ***et al.*** ** (39)**	Assam	100	5 (5)	4	6	3
**Ambulkar ** ***et al.*** ** (40)**	Wardha	58	6 (10.7)	10	6	10.7
**Sathyanarayana ** ***et al.*** ** (41)**	Mysore	200	1 (0.50)	6	2	0
**Rani ** ***et al.*** ** (33)**	Hyderabad	973	286 (29.4)	35	6	16.4
**Present study (2019)**	Mangalore DK	100	2 (2)	10	6	2
DK: Dakshina kannada, EAA: European academy of andrology, STS: Sequence-tagged sites						

## 5. Conclusion

The present study emphasizes the importance of routine cytogenetic screening of all infertile males before undergoing ART. It is not only to identify the underlying cause of infertility but also to minimize the risk of transmission of chromosomal abnormalities. Infertile males with abnormal semen parameters should undergo screening of Y microdeletion before the treatment to reducing the transmission of the same abnormality to the offspring. When the male partner has an abnormal autosomal karyotype abnormality, genetic counseling should be offered to the couple seeking fertility treatment. The observed results would give a better understanding of the etiology and particular phenotype of male infertility. To the best of our knowledge, this study reports for the first time a case with unique, balanced carrier translocation of chromosome 2q21 and 7p21. Cytogenetic and Y microdeletion screening would provide specific and better treatment options during genetic counseling of infertile male.

##  Conflict of Interest 

The authors declare that they have no conflict of interest.
